# Characterizing Heterogeneity in Behavioral Phenotypes Among Oral Nicotine Pouch Users in Saudi Arabia: A Latent Class Analysis

**DOI:** 10.7759/cureus.106912

**Published:** 2026-04-12

**Authors:** Abdullah M Gosadi, Naif A Dighriri, Yahya A Hurubi, Abdulkarim H Safhi, Ali M Mujarribi, Fahad M Mashyakhi, Ahmed M Hodani, Abdullah A Ageeli, Albara E Tayran, Abdulmjeed H Somily, Nawaf A Darbashi, Ayah H Alghamadi, Hassan A Madkhali, Fahad Y Sabei

**Affiliations:** 1 College of Pharmacy, Jazan University, Jazan, SAU; 2 College of Medicine, Jazan University, Jazan, SAU; 3 College of Pharmacy, King Abdulaziz University, Jeddah, SAU; 4 Department of Pharmaceutics, College of Pharmacy, Jazan University, Jazan, SAU

**Keywords:** adverse effects, harm reduction, latent class analysis, nicotine dependence, oral nicotine pouches, poly-tobacco use, risk profiles, saudi arabia, smokeless tobacco

## Abstract

Background

The rapid proliferation of oral nicotine pouches has polarized public health discourse, framing these products as either reduced-risk alternatives or catalysts for novel addiction pathways. Conventional research frequently obscures the functional heterogeneity underlying these outcomes. This investigation employs a person-centered framework to identify distinct behavioral phenotypes among adult users in Saudi Arabia and evaluate their clinical implications.

Methods

A cross-sectional analytical study assessed adult pouch consumers (N=310) residing in the Jazan province. Latent class analysis-derived user typologies are mathematically grounded in behavioral indicators, encompassing consumption frequency, nicotine strength preference, poly-product utilization, and functional motives.

Results

The analysis revealed three divergent trajectories. The "Harm Reduction-Motivated De-escalator" phenotype (n=65, 21.0%) exhibited low-intensity consumption driven by tapering intent. Conversely, the "Stable, Affect-Regulating Dual-User" (n=117, 37.7%) displayed a maintenance pattern characterized by homeostatic mood management. Most critically, the "Escalating, High-Intensity Poly-Product User" (n=128, 41.3%) emerged as a high-risk cohort defined by a distinct preference for supratherapeutic nicotine strengths (≥10 mg) alongside the highest rate of simultaneous poly-product use (n=67, 52.3%). Consequently, this escalating group demonstrated significantly elevated odds of experiencing localized gum irritation (aOR = 3.25) compared to the de-escalator baseline.

Conclusions

Consumers do not appear to constitute a monolithic population; clinical risk appears to be shaped primarily by user behaviors and functional motivations rather than inherent product properties alone. Although a minority utilizes these products for rational harm reduction, the concerning prevalence of escalating poly-users highlights a potential toxicity trap driven by high-intensity stacking behaviors. Public health strategies might benefit from transitioning toward targeted behavioral profiling and customized therapeutic interventions.

## Introduction

Oral nicotine pouches (ONPs) have emerged as a disruptive innovation in the global nicotine market, decoupling nicotine delivery from the combustion-derived toxicants of traditional tobacco. This shift is facilitated by specific formulation strategies, notably the use of alkaline pH adjusters that maximize the unprotonated, bioavailable fraction of nicotine [1,2]. While the absence of tobacco-specific nitrosamines (TSNAs) and polycyclic aromatic hydrocarbons supports a potential role in harm reduction [3,4], the "tobacco-free" designation obscures a complex toxicological reality. Recent in vitro assays indicate that non-nicotine constituents, specifically flavorings and synthetic coolants, can induce dose-dependent mitochondrial dysfunction and inflammatory cytokine release in human gingival fibroblasts, suggesting mechanisms for local tissue injury that exist independently of systemic nicotine exposure [5-7].

This toxicological complexity is paralleled by a distinct epidemiological pattern in the Gulf Cooperation Council (GCC) region. In contrast to the youth-driven "gateway" trajectories often observed in North American and European surveillance [8,9], emerging data from Saudi Arabia point to a functional etiology characterized by high-intensity "stacking" among established adult users. Local prevalence studies reveal that up to 95.8% of ONP users are concurrent smokers, a finding that aligns with a "Common Liability" model where ONPs may serve as an adjunctive source of neuroadaptive maintenance [10-12]. This high rate of poly-product use raises concerns that users may be accumulating systemic nicotine loads that exceed the saturation limits of exclusive smoking, potentially driving the high rates of gastrointestinal and autonomic adverse effects reported in the region [13,14].

The unique pharmacokinetic profile of ONPs, which exhibits a delayed Tmax and blunted Cmax compared to combustible cigarettes [15], presents a challenge for clinical assessment. Standard psychometric tools, such as the Fagerström Test for Nicotine Dependence (FTND), were originally calibrated for the rapid arterial bolus of smoking. Given the steady-state kinetics of oral pouches, it remains unclear whether these traditional metrics possess the construct validity to accurately capture dependence in modern poly-product users, or if they underestimate severity in those exhibiting high behavioral consumption without acute withdrawal urgency [16,17]. Consequently, evaluating the utility of the FTND in this specific population is a critical step toward establishing appropriate surveillance standards.

To fully characterize these usage patterns, there is a need to move beyond aggregate statistics to explore the underlying heterogeneity of the user population. As noted by Nylund-Gibson and Choi [18], person-centered approaches such as latent class analysis (LCA) allow for the identification of unobserved subgroups or "typologies" that variable-centered averages may obscure. This study applies LCA to the Jazan province context to empirically derive distinct ONP user phenotypes based on consumption behaviors and motivations. Specifically, the objectives of this study were threefold: (1) to empirically derive distinct behavioral phenotypes among adult ONP users in the Jazan province using LCA; (2) to evaluate whether these phenotypes differ in their associations with nicotine dependence severity, consumption volume, and self-reported adverse effects; and (3) to assess the construct utility of the adapted Fagerström Test for Nicotine Dependence in this novel poly-product context. By mapping these profiles, we aim to provide a more nuanced understanding of ONP use risks, thereby informing more targeted public health and clinical strategies.

## Materials and methods

Study design and participants

We employed a cross-sectional, analytical design to survey adults in the Jazan province of Saudi Arabia between March 17, 2025, and July 10, 2025. To be considered for inclusion, individuals were required to be at least 18 years of age in addition to having used ONPs at least once during the preceding 30 days. Individuals presenting a self-reported history of chronic diseases that could potentially confound the assessment of adverse effects were excluded. Recruitment was conducted through a non-probability purposive sampling strategy. This approach was further supplemented with snowball sampling techniques to effectively reach what is often a specialized population of users. From an initial pool of respondents, 80 individuals were removed prior to analysis. Specifically, 50 respondents were excluded for failing to meet the past-30-day usage criterion. Another 20 individuals reported pre-existing chronic conditions that warranted removal, while an additional 10 participants simply did not finalize the survey. These exclusions yielded a final analytical sample of 310 participants.

Sample size requirements in latent class modeling do not adhere to a singular universal formula. Rather, adequate powering seems to depend heavily upon the degree of class separation alongside the specific quality of the selected indicators. To appropriately plan our analytical framework, we relied upon simulation-based methodological guidelines. Research by Dziak and colleagues suggests that achieving an 80% statistical power threshold for a three-class model with five indicators is often possible utilizing approximately 200 observations, assuming at least a moderate degree of class separation [19]. Furthermore, Sinha and co-authors have indicated that this modeling approach generally performs optimally as a large-sample methodology. Their syntheses imply that model selection indices tend to become unreliable when participant numbers fall below the 300 threshold, which often necessitates bespoke Monte Carlo simulations for validation in those restricted cohorts [20]. Consequently, our final attained sample of 310 individuals was considered sufficiently robust. It safely clears the potentially problematic small-sample boundary while appearing to provide adequate mathematical power for reliable class enumeration.

Procedure and measures

A structured electronic survey was developed and administered in both Arabic and English. The instrument underwent a standard forward-backward translation protocol [21] to establish conceptual and linguistic equivalence. This process was followed by face and content validation conducted by a panel of public health and tobacco research experts [22]. A subsequent pilot test involving a small group of 25 ONP users appeared to confirm the instrument's clarity and general usability.

The finalized survey captured data across four primary domains. Initial sections gathered sociodemographic characteristics alongside detailed nicotine use histories. This historical data encompassed the age of initiation as well as the concurrent utilization of alternative products. Subsequent survey modules were dedicated to capturing behavioral and motivational indicators for ONP consumption, ultimately concluding with a comprehensive assessment of clinical outcomes. For the primary analysis, the behavioral and motivational metrics were operationalized as six distinct binary variables. These indicators captured poly-nicotine use alongside a distinct preference for high-strength products containing 10 mg or more of nicotine. The analytical variables additionally tracked reported consumption increases over the past six months as well as intensive simultaneous pouch use. Finally, the models incorporated sustained motivations explicitly related to both affect regulation and harm reduction.

Within the clinical outcome domain, nicotine dependence was evaluated via the FTND. This instrument was originally developed to refine preceding tolerance questionnaires specifically for cigarette smokers [23]. We utilized the smokeless tobacco adaptation of this established scale. That particular version was initially validated to bypass the necessity for users to rate product nicotine content, a limitation frequently encountered when assessing alternative nicotine delivery systems [24]. Our further modification involved substituting the term "smokeless tobacco" with "oral nicotine pouches" across all relevant prompts without fundamentally altering the underlying psychological construct. The scoring mechanics remained identical to standard diagnostic protocols. Two specific questions are evaluated on a scale from zero to three, whereas the remaining four items utilize a binary zero-to-one format. Summing these values yields a potential maximum score of ten. Clinical interpretation thresholds generally classify a score of one to two as indicative of low dependence. Scores ranging from three to four may suggest low to moderate dependence, while a result of five to seven could potentially be interpreted as moderate. Any total reaching eight or above is typically regarded as reflecting high dependence. The modified scale demonstrated acceptable internal consistency in this current cohort, yielding a raw Cronbach’s α of 0.65 alongside a standardized Cronbach’s α of 0.70. Additionally, the average inter-item correlation was 0.32. This value falls within what is often considered an optimal range, appearing to support the structural reliability of the adapted metric. The experience of nine specific adverse effects was subsequently assessed using a 5-point frequency scale ranging from 0 ("Never") to 4 ("Frequently").

Statistical analysis

All statistical evaluations were conducted utilizing R statistical software [25]. Initial variable-centered regression models designed to predict consumption volume yielded relatively low explanatory power. This empirical observation suggested the presence of profound unobserved heterogeneity within the user population, which mandated a pivot toward a person-centered analytical framework as the primary approach for this study.

Latent Class Analysis, a model-based clustering technique designed to identify unobserved subgroups, was employed to derive user phenotypes [18, 26]. Models were estimated for two through five classes utilizing Mplus [27] via the MplusAutomation package in R. All models were estimated using maximum likelihood with robust standard errors (MLR) and employed 500 random start values (with 50 optimizations carried through to completion) to mitigate the risk of convergence on local maxima. The six binary indicator variables entered into the LCA were coded as follows: poly-nicotine use (use of ONPs plus more than one additional nicotine product = 1, otherwise = 0), high-strength preference (primary use of ≥10 mg products = 1, otherwise = 0), increased consumption over the past six months (self-reported increase = 1, otherwise = 0), intensive simultaneous use (weekly or almost daily use of multiple pouches at once = 1, otherwise = 0), affect regulation motive (endorsed = 1, not endorsed = 0), and harm reduction motive (endorsed = 1, not endorsed = 0). Local independence of indicators conditional on class membership was assumed, consistent with standard LCA practice. The three-class solution was ultimately selected because it appeared to provide the most optimal balance of statistical fit (demonstrating the lowest Akaike Information Criterion) and robust classification quality, while generating theoretical profiles that were distinct and highly interpretable. To properly account for the probabilistic nature of latent modeling and minimize potential classification errors, a posterior-probability weighting method was applied throughout all subsequent analyses [28]. This weighting was implemented via the survey package in R [29].

The potential association between latent class membership and various clinical outcomes was investigated using a series of multivariable regression models, which were adjusted to control for sociodemographic confounders. For the ordinal FTND score as well as the nine individual adverse effect frequency outcomes, a proportional odds ordinal logistic regression was applied, as it serves as the standard approach for ordered categorical data [30]. A negative binomial regression was specified to accommodate the overdispersed count of weekly pouch consumption [31]. Finally, to analyze the zero-inflated total adverse effects count, a two-part hurdle negative binomial model was employed. This allowed us to separately model the probability of experiencing any adverse effect versus none at all, alongside the expected count of effects among those symptomatic users [32-34].

Ethical considerations

Ethical approval was granted by the Standing Committee for Scientific Research at Jazan University (approval reference number: REC-47/01/1563; HAPO-10-Z-001). All participants provided informed electronic consent prior to inclusion in the study, and all data were collected and stored anonymously.

## Results

Latent class analysis fit & diagnostics

To empirically characterize the underlying behavioral heterogeneity within the analytical cohort, a series of latent class models specifying two through five unobserved subgroups was systematically estimated. Evaluating the comparative fit indices suggests that a three-class structural configuration might offer the most parsimonious balance between statistical robustness and theoretical interpretability. As delineated in Table 1, the transition from a two-class to a three-class parameterization yielded an observable reduction in both the Akaike Information Criterion, dropping from 3173.51 to 3166.567, and the sample-size adjusted Bayesian Information Criterion, which decreased from 3185.374 to 3184.645. Although the four-class and five-class models exhibited continued improvements in log-likelihood estimations (-1540.769 and -1530.746, respectively), these more complex specifications concurrently generated penalty-driven escalations in the Bayesian Information Criterion, rising to 3328.211 and 3371.267. Furthermore, the three-class solution demonstrated a notable enhancement in classification entropy (0.685) relative to the two-class baseline (0.439), an outcome that generally denotes improved boundary differentiation among the derived typologies. Consequently, this tri-partite model was provisionally retained, as it appears to adequately encapsulate the data structure without risking the over-extraction typically inherent to higher-order solutions.

**Table 1:** Model Fit Statistics for Latent Class Analysis (LCA) Solutions Fit statistics are presented for models with two through five latent classes. The three-class solution was selected as the optimal model based on a holistic evaluation of these indices, prioritizing the lowest Akaike Information Criterion (AIC), a substantial improvement in Entropy over the two-class solution, and strong theoretical interpretability. AIC, Akaike Information Criterion; BIC, Bayesian Information Criterion; aBIC, Sample-Size Adjusted BIC; LL, Log-likelihood.

Model Fit Statistic	Two-Class Model	Three-Class Model	Four-Class Model	Five-Class Model
Log-likelihood (LL)	-1565.755	-1551.283	-1540.769	-1530.746
AIC	3173.51	3166.567	3167.538	3169.492
BIC	3251.978	3286.137	3328.211	3371.267
Sample-Size Adjusted BIC (aBIC)	3185.374	3184.645	3191.831	3199.999
Entropy	0.439	0.685	0.676	0.674

Following the provisional selection of the three-class architecture, subsequent diagnostic evaluations were conducted to appraise the precision of the probabilistic participant assignments. An examination of the posterior classification metrics, presented in Table 2, reveals that the adopted model seems to exhibit substantial assignment certainty. Specifically, the average posterior probabilities for the emergent profiles, representing approximately 20.9%, 37.7%, and 41.4% of the cohort respectively, were calculated at 0.887 for the first class, 0.806 for the second, and 0.861 for the third. Given that these estimations uniformly surpass the conventional adequacy threshold of 0.80, the intra-class homogeneity is considered potentially robust. Coupled with the overarching entropy coefficient of 0.685, which implies a reasonable degree of separation between the estimated latent strata, the derived phenotypic weighting distributions are deemed sufficiently reliable to facilitate the subsequent adjusted analytical phases of this investigation.

**Table 2:** Classification Diagnostics and Posterior Probability Estimates for the Adopted Three-Class Model This table presents the final classification diagnostics for the selected three-class solution. N represents the raw count of participants modally assigned to each class. Proportion is the percentage of the total sample. The average posterior probability (APP) is a measure of classification certainty, representing the average probability of correct classification for members of a class. An APP ≥ 0.80 is considered to indicate strong classification quality.

Classification Metric	Class 1	Class 2	Class 3	Overall Model
N	65	117	128	
Proportion	0.209	0.377	0.414	
Avg. Posterior Prob (APP)	0.887	0.806	0.861	
Entropy				0.685

**Figure 1:**
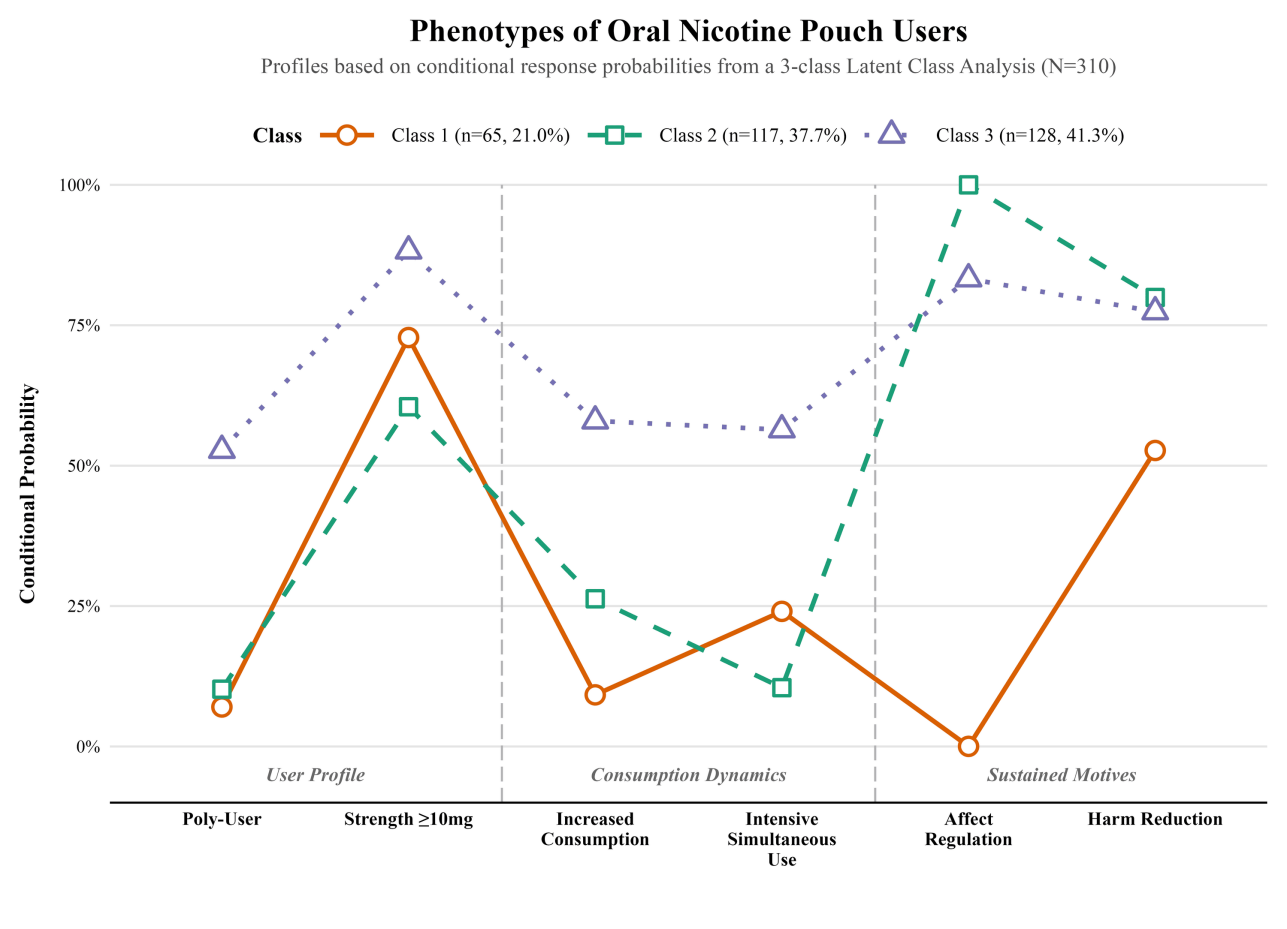
Conditional Response Probabilities Characterizing the Identified Oral Nicotine Pouch User Typologies Profiles are based on conditional response probabilities from a three-class latent class analysis (N=310). The y-axis represents the probability of a member of a given class endorsing the specific behavioral or motivational indicator shown on the x-axis. Indicator definitions are as follows: Poly-User indicates use of ONPs plus more than one other nicotine product. Strength ≥10mg indicates a preference for high-strength pouches. Increased Consumption indicates a self-reported increase in use over the past six months. Intensive Simultaneous Use indicates a response of 'Frequently (weekly)' or 'Regularly (almost daily)' to using multiple pouches at once. Affect Regulation and Harm Reduction indicate endorsement of these items as sustained motives for use. ONP, Oral Nicotine Pouch

Building upon the probabilistic assignments established in Table 2, the defining behavioral and motivational characteristics of the three emergent typologies are visually delineated within Figure 1. The initial phenotype, tentatively designated as Harm Reduction-Motivated De-escalators, comprised approximately 21% of the analytical cohort and was predominantly characterized by a comparatively high probability of having reduced ONP consumption over the preceding six months. Furthermore, individuals clustered within this stratum exhibited a notable propensity to endorse harm reduction as a primary sustained motive, concurrently displaying a near-zero probability of utilizing the products for affective moderation.

Conversely, the second profile, representing 38% of the sample and labeled as Stable Affect-Regulating Dual-Users, was uniquely distinguished by a universal conditional probability of endorsing affect regulation. This specific subgroup appeared to be largely populated by dual-users who reported relatively stable consumption dynamics, coupled with the lowest overall likelihood of preferring high-strength formulations.

Finally, the third and most prominent classification, encompassing roughly 41% of participants, was categorized as Escalating High-Intensity Poly-Product Users. This particular phenotype demonstrated a near-universal preference for products containing 10 milligrams or more of nicotine, alongside the highest probabilities for concurrent poly-product utilization, increased consumption trajectories, and the frequent simultaneous application of multiple pouches.

Sociodemographic and nicotine use profile of the cohort

The final analytical cohort consisted of 310 adults who use ONPs. The overall sample was predominantly young (median age = 24, IQR [23,28]), male (n=302, 97.4%), and characterized by high rates of concurrent nicotine use, with 80.0% (n=247) of participants being either dual- or poly-product users. As detailed in Table 3, weighted analyses revealed no statistically significant differences across the three emergent latent classes for sociodemographic characteristics, including age (p=0.6), gender (p=0.4), and educational level (p=0.4). The classes were, however, significantly different regarding the behavioral and motivational indicators used for their classification, such as user type (p<0.001) and preference for high-strength ONPs (≥ 10mg) (p<0.001).

**Table 3:** Weighted Sociodemographic and Nicotine Use Characteristics of the Study Cohort, Stratified by Latent Class Phenotype Data are presented as median (Interquartile Range) for continuous variables and n (weighted %) for categorical variables. The total sample size (N) reflects the number of individual participants. Class sizes in the column headers represent the weighted population estimates derived from the latent class analysis (LCA) posterior probabilities. Test statistics and corresponding p-values are derived from weighted complex survey analyses: a design-based rank test (Kruskal-Wallis equivalent) for continuous variables and a Rao-Scott adjusted chi-square test for categorical variables. Effect Size abbreviations: SMD, Standardized Mean Difference (for continuous and binary variables); V, Cramér's V (for multi-category variables). † Note on Perfect Separation: The weighted test statistic for "Sustained Motive: Affect" was not computed due to perfect separation between classes (i.e., zero variance within the stratum, where the variable perfectly predicts class membership), precluding the calculation of a standard Rao-Scott adjusted chi-square matrix.

Characteristic	Overall N = 310	Class 1 N = 65	Class 2 N = 117	Class 3 N = 128	Test Statistic Value	p-value^*^	Effect Size
Age	24 (23, 28)	25 (23, 28)	24 (22, 28)	25 (22, 28)	1.09	0.6	SMD = 0.162
Gender					4.78	0.4	SMD = 0.157
Female	8 (2.6%)	1 (1.5%)	5 (4.0%)	2 (1.8%)			
Male	302 (97%)	64 (98%)	112 (96%)	126 (98%)			
Educational Level					3.91	0.4	SMD = 0.177
High School or Less	104 (34%)	18 (28%)	43 (36%)	43 (34%)			
Bachelor's or Higher	206 (66%)	47 (72%)	74 (64%)	85 (66%)			
Occupation					4.88	0.7	V = 0.089
Employed	158 (51%)	30 (47%)	61 (52%)	67 (52%)			
Unemployed/Other	51 (16%)	12 (18%)	16 (14%)	23 (18%)			
Student	101 (33%)	23 (35%)	40 (34%)	38 (30%)			
Residence					0.31	>0.9	SMD = 0.049
Rural	134 (43%)	29 (45%)	51 (43%)	54 (42%)			
Urban	176 (57%)	36 (55%)	66 (57%)	74 (58%)			
Monthly Income (SAR)	5,000 (1,000, 9,169)	4,500 (1,000, 8,700)	5,000 (1,000, 9,600)	5,000 (1,000, 10,000)	1.16	0.6	SMD = 0.197
User Type					218.72	<0.001	V = 0.594
Exclusive ONP User	63 (20%)	17 (26%)	28 (24%)	18 (14%)			
Dual-user	163 (53%)	43 (67%)	77 (66%)	43 (33%)			
Poly-user	84 (27%)	5 (7.0%)	12 (10%)	67 (53%)			
ONP High Strength (≥ 10mg)	231 (75%)	47 (73%)	71 (60%)	113 (88%)	74.87	<0.001	SMD = 0.637
Change in ONP Consumption Over Past Six Months					169.53	<0.001	V = 0.523
Decreased (less frequent)	85 (27%)	31 (47%)	31 (27%)	23 (18%)			
Stayed the same	114 (37%)	28 (44%)	55 (47%)	31 (24%)			
Increased (more frequent)	111 (36%)	6 (9.2%)	31 (26%)	74 (58%)			
Frequency of Simultaneous Pouch Use					265.1	<0.001	
Regularly (almost daily)	44 (14%)	10 (15%)	12 (10%)	22 (17%)			
Frequently (weekly)	56 (18%)	6 (9.0%)	0 (<0.1%)	50 (39%)			
Occasionally (once or twice a month)	127 (41%)	22 (35%)	60 (52%)	44 (34%)			
Never	83 (27%)	27 (41%)	45 (38%)	12 (9.1%)			
Sustained Motive: Affect	224 (72%)	0 (<0.1%)	117 (100%)	107 (83%)	†	PS	SMD = 2.232
Sustained Motive: Convenience	247 (80%)	37 (57%)	101 (87%)	108 (85%)	79.42	<0.001	SMD = 0.744
Sustained Motive: HR	227 (73%)	34 (53%)	93 (80%)	99 (77%)	53.21	<0.001	SMD = 0.615

Phenotypic correlates of nicotine dependence and clinical outcomes

To delineate the clinical implications of the derived user typologies, an initial evaluation of unadjusted bivariate associations between latent class membership and varying clinical indices was conducted. As detailed in Table 4, preliminary analyses indicated that median weekly pouch consumption appeared to be significantly elevated among individuals classified within the third phenotype, yielding a median of 28 pouches compared to 20 for the first class (p=0.039). Furthermore, a crude metric of subjective reliance, designated as a "Dependence Proxy (Necessity)", was integrated to capture the users' self-perceived requirement for daily ONP consumption based on a direct questioning of their perceived need. This subjective assessment suggested a statistically significant variance across the strata (p=0.009), with the third and second classes reporting higher median necessity scores relative to the reference group. Additionally, the unadjusted distribution of the total adverse effects count pointed toward a potential divergence, wherein the third class exhibited a higher median occurrence (p=0.043). Conversely, the raw FTND scores did not manifest a statistically significant differentiation across the three profiles at this unadjusted analytical stage (p=0.14).

**Table 4:** Unadjusted Bivariate Correlates Between Latent Typologies and Preliminary Clinical Endpoints Data are presented as median (interquartile range). Clinical and consumption outcomes were compared across the three latent classes using a design-based Kruskal-Wallis rank test to account for the weighted data. The reported Test Statistics represent the chi-square (𝝌2) approximation with 2 degrees of freedom for all variables. All p-values are derived from these weighted statistical tests. † Dependence Proxy (Necessity) represents a subjective assessment of the user's perceived reliance on oral nicotine pouches. It is a composite score calculated from two reverse-coded survey items: "Over time, I have started to feel that ONPs are unnecessary for me" and "I could stop using ONPs anytime I wanted to, without any difficulty”. ONPs, Oral Nicotine Pouches

Clinical Outcome	Class 1 N = 65	Class 2 N = 117	Class 3 N = 128	Tests Statistic (𝝌^2^)	Weighted p-value
Fagerström Score (FTND)	3 (1, 5)	3 (1, 5)	4 (2, 6)	4.005	0.14
Weekly Pouch Consumption	20 (9, 35)	21 (9, 42)	28 (12, 42)	6.583	0.039
Dependence Proxy (Necessity)†	2 (2, 3)	3 (2, 3)	3 (2, 4)	9.516	0.009
Total Adverse Effects Count	20 (8, 34)	20 (8, 30)	26 (12, 36)	6.381	0.043

Recognizing that crude bivariate observations might be confounded by underlying sociodemographic heterogeneities, a subsequent series of multivariable regression models was specified to isolate the adjusted effect of phenotypic classification on the aforementioned clinical endpoints. Table 5 presents the estimations derived from a proportional odds ordinal logistic regression, which was operationalized to assess the adapted FTND. Upon adjusting for requisite covariates, the relationship between latent class affiliation and structural nicotine dependence severity was seemingly attenuated. Specifically, membership in the high-intensity third class, when contrasted against the de-escalating reference cohort, did not reliably predict a higher ordinal dependence score (aOR = 1.33, 95% CI [0.78, 2.28], p=0.3). This statistical attenuation potentially implies that conventional psychometric evaluations of physical dependence might lack the construct sensitivity required to accurately differentiate between high-volume poly-product users and those exhibiting lower, tapering consumption trajectories.

**Table 5:** Adjusted Association Between Latent Class and Nicotine Dependence (FTND Score): Ordinal Regression Model Model: Proportional odds ordinal logistic regression evaluating the association between latent class membership and adapted FTND score. Adjusted covariates: Age, educational attainment (Bachelor's degree or higher vs. ≤ HS), occupational status, log-transformed monthly income, and residential setting. Reference category: Class 1. aOR, Adjusted Odds Ratio; CI, Confidence Interval; FTND, Fagerström Test for Nicotine Dependence; HS, High School.

Characteristic	aOR	95% CI	p-value
Latent Class			
Class 2 (vs. Class 1)	1.12	0.67, 1.89	0.7
Class 3 (vs. Class 1)	1.33	0.78, 2.28	0.3
Age	0.95	0.92, 0.98	0.002
Education: Bachelor's+ (vs. ≤ HS)	0.86	0.64, 1.15	0.3
Occupation			
Unemployed/Other (vs. Employed)	1.69	0.78, 3.64	0.2
Student (vs. Employed)	1.05	0.52, 2.10	0.9
Log Monthly Income	1.14	0.82, 1.60	0.4
Residence: Urban (vs. Rural)	1.23	0.82, 1.85	0.3

In parallel with the dependence metrics, a negative binomial regression analysis was conducted to elucidate the adjusted relationship between the latent typologies and the overdispersed count of weekly pouch consumption, as presented in Table 6. Following the inclusion of sociodemographic covariates, the ostensibly higher consumption volume observed in the unadjusted third class was reduced to a non-significant trend. Specifically, members of the escalating, high-intensity poly-product user phenotype exhibited an estimated 27% increase in the rate of weekly pouch utilization relative to the harm reduction-motivated de-escalators (aIRR = 1.27, 95% CI [0.98, 1.64], p=0.064). This attenuation suggests that variations in raw consumption magnitude might be partially attributable to confounding demographic factors, such as age and monthly income, rather than being exclusively driven by the underlying behavioral phenotype itself.

**Table 6:** Adjusted Association Between Latent Class and Weekly Pouch Consumption: Negative Binomial Model Model: Negative binomial regression evaluating the association between latent class membership and overdispersed weekly pouch consumption count. Adjusted covariates: Age, educational attainment (Bachelor's degree or higher vs. ≤ HS), occupational status, log-transformed monthly income, and residential setting. Reference category: Class 1. CI, Confidence Interval; HS, High School; IRR, Incidence Rate Ratio.

Characteristic	IRR	95% CI	p-value
Latent Class			
Class 2 (vs. Class 1)	1.17	0.91, 1.49	0.2
Class 3 (vs. Class 1)	1.27	0.98, 1.64	0.064
Age	0.97	0.95, 0.98	<0.001
Education: Bachelor's+ (vs. ≤ HS)	1.02	0.88, 1.17	0.8
Occupation			
Unemployed/Other (vs. Employed)	1.26	0.86, 1.87	0.2
Student (vs. Employed)	0.93	0.68, 1.29	0.7
Log Monthly Income	1.20	1.02, 1.41	0.027
Residence: Urban (vs. Rural)	0.97	0.80, 1.19	0.8

To comprehensively evaluate the generalized toxicological burden associated with these distinct usage patterns, a two-part hurdle negative binomial model was initially utilized to analyze the aggregate count of self-reported side effects. The adjusted multivariable analysis indicated that phenotypic classification did not reliably predict the fundamental susceptibility to experiencing any initial adverse symptoms. Furthermore, among those individuals who reported a minimum of one symptom, the truncated count component suggested only a marginal, non-significant trend toward a higher cumulative burden for the third class relative to the reference group (aIRR = 2.83, 95% CI [0.87, 9.21], p=0.084).

**Figure 2:**
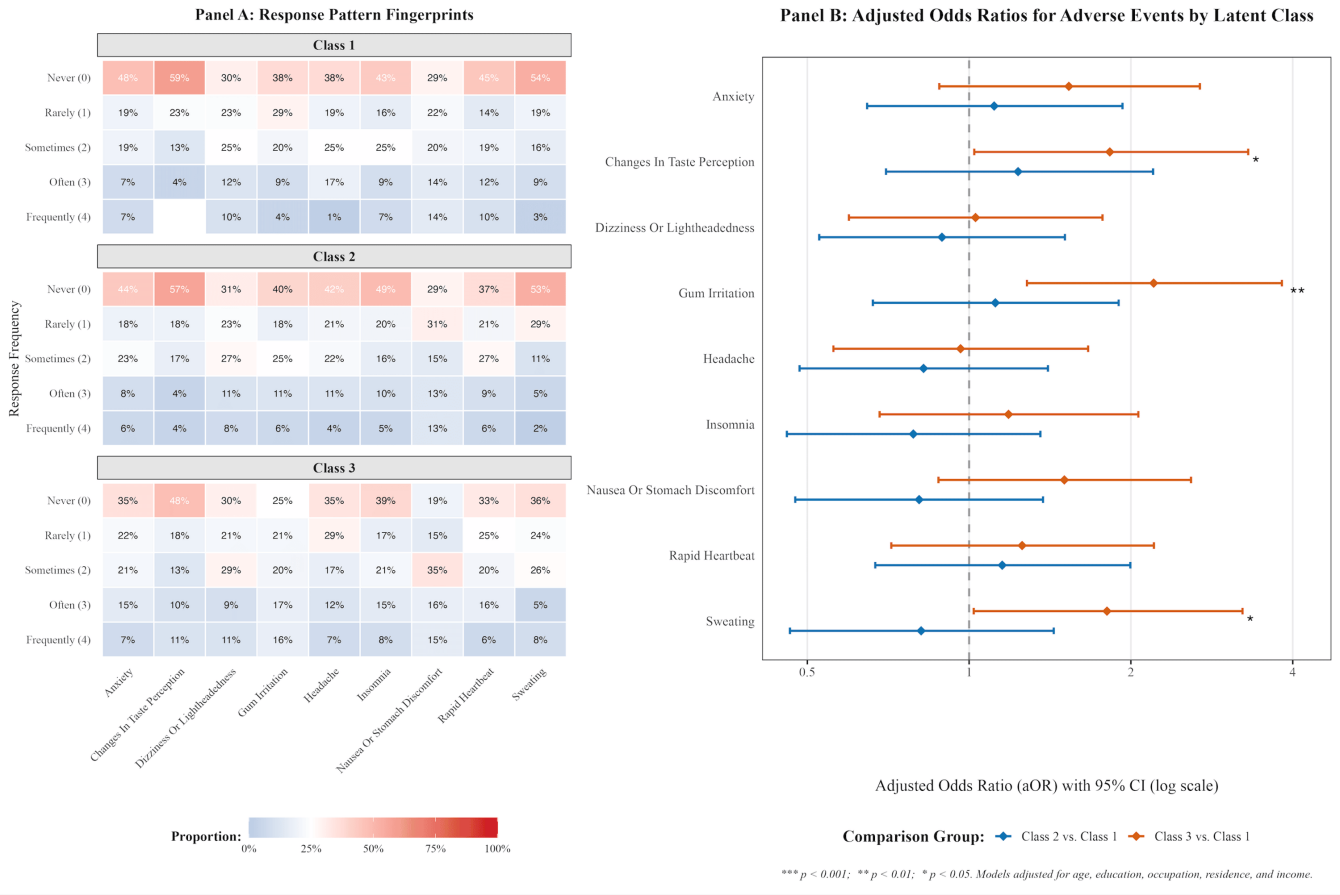
Multidimensional Assessment of Adverse Event Profiles Stratified by User Phenotype Panel A provides heatmaps delineating the response distributions for nine self-reported adverse manifestations across the latent classes (Class 1, n=65; Class 2, n=117; Class 3, n=128), with color intensity corresponding to endorsement frequency. Panel B visualizes the adjusted odds ratios (aORs), represented on a logarithmic scale, alongside their 95% confidence intervals, derived from multivariable ordinal logistic regression models. These models illustrate the odds of reporting elevated symptom frequencies for Class 2 and Class 3 relative to the Class 1 reference cohort, adjusting for pertinent sociodemographic variables. *p < 0.05; ** p < 0.001.

Given the lack of statistical robustness in the aggregate symptom volume, subsequent analyses were directed toward evaluating the frequency distributions of nine specific adverse manifestations, as delineated in Figure 2. Utilizing adjusted proportional odds ordinal logistic regression models, membership in the escalating high-intensity poly-product user typology was observed to correlate with a seemingly exacerbated risk profile for localized oral and specific systemic symptoms.

Most notably, when contrasted against the harm reduction-motivated de-escalator reference cohort, individuals classified within the third phenotype exhibited over three times the odds of reporting more frequent occurrences of gum irritation (aOR = 3.25, 95% CI [1.89, 5.58], p < 0.001). Additionally, this high-intensity subgroup demonstrated statistically significant elevations in the likelihood of experiencing altered gustatory sensations, specifically changes in taste perception (aOR = 1.82, 95% CI [1.02, 3.17], p=0.042), alongside increased frequencies of diaphoresis or sweating (aOR = 1.80, 95% CI [1.02, 3.23], p=0.044). These targeted findings potentially underscore that while the overall multiplicity of adverse events might remain relatively uniform across the user spectrum, the specific pathophysiological consequences of aggressive poly-product stacking appear to be heavily concentrated in localized mucosal irritation and specific autonomic responses.

## Discussion

The application of a person-centered LCA in this study investigates Saudi ONP users beyond the "average" by empirically isolating three distinct functional etiologies within a single population. While global surveillance often depicts ONP use as a monolithic youth trend [8,9], our data reveals a complex adult ecosystem where risk is determined not by the product, but by the user’s neuroadaptive trajectory. The identification of three discrete phenotypes: "Harm Reduction-Motivated De-escalators" (Class 1), "Stable, Affect-Regulating Dual-Users" (Class 2), and "Escalating, High-Intensity Poly-Product Users" (Class 3) provides the first granular evidence that the Saudi market is simultaneously a landscape of rational harm reduction and profound toxicological escalation.

The "Rational Switcher" phenotype (Class 1, 21%) offers strong empirical validation for the harm reduction potential of ONPs within the Jazan context. This group’s defining characteristics, low daily consumption (<5 pouches), preference for lower nicotine strengths (<6 mg), and a 47% probability of decreasing use, are consistent with the "successful switcher" profile identified in recent Saudi cessation data [11]. Mechanistically, one possible explanation for this de-escalation is the specific pharmacokinetic profile of standard-strength pouches used by this class. As demonstrated by Kanobe et al. [15], lower-dose pouches exhibit a delayed Tmax (30 min) and blunted Cmax that mimic the steady-state kinetics of nicotine replacement therapy (NRT) rather than the arterial bolus of cigarettes. By utilizing these "NRT-mimics", Class 1 users effectively manage withdrawal without triggering the reinforcement sensitization that drives escalation, a conclusion supported by chemical analyses confirming the absence of TSNAs in these products [3,4].

In contrast, the "Stable, Affect-Regulating Dual-User" (Class 2, 38%) represents a phenotype driven by functional relief rather than dependence-driven escalation. The defining feature of this class, a 100% endorsement of "Affect Regulation" as a primary motive combined with stable consumption patterns, validates Hogarth’s theory of "Goal-Directed Drug Choice" [35]. Unlike the compulsive user, this phenotype appears to titrate nicotine intake to a homeostatic set-point required to mitigate underlying emotion regulation deficits, a vulnerability prevalent in substance-using populations [36]. Neural imaging studies suggest that these users are likely leveraging nicotine’s capacity to desensitize amygdala circuits [37] and enhance attentional orienting [38,39] to treat "cognitive withdrawal" [40]. Their stability is therefore not a transient phase, but a sustained "balance mode" [41] that persists as long as the affective deficit requires management.

The most clinically critical finding is the "Toxicity Trap" characterizing Class 3 (41%), where behavioral escalation directly translates into biological pathology. This group’s aggressive profile, 88% preference for high-strength pouches (≥10 mg) and 53% poly-product use, reflects a collision between high tolerance and the "Pharmacokinetic Mismatch" of modern pouches. While standard pouches lack a bolus [15], the high-dose variants preferred by this class deliver a massive total nicotine load (AUC) that can exceed cigarettes [42,43]. This necessitates a formulation chemistry relying on high concentrations of artificial sweeteners to mask bitterness [44] and alkaline pH adjusters to force absorption [1]. Consequently, the significantly elevated odds of gum irritation (aOR = 3.25) observed in this class are biologically consistent with in vitro evidence of flavorant-induced cytotoxicity [6] and pH-driven mucosal lesions [45,46]. Furthermore, the high prevalence of systemic symptoms suggests that these "stackers" are exceeding their enzymatic clearance capacity, resulting in cholinergic toxicity [13].

Finally, the statistical failure of the FTND-ST to distinguish this high-risk Class 3 from the low-risk Class 1 (p=0.3) exposes a fundamental measurement gap. The FTND is calibrated to measure "physical urgency" (e.g., time to first cigarette) driven by rapid-clearance combustion kinetics. It lacks the construct validity to detect the "situational stacking" behavior [17] of Class 3 users, who maintain high serum nicotine levels through poly-use and therefore rarely experience the acute withdrawal urgency the test is designed to measure. This finding, reinforced by the systematic review by Sharma et al. [16] citing the tool’s unacceptable internal consistency (ɑ< 0.70) in novel product users, confirms that standard psychometrics are "blind" to the high-tolerance, steady-state dependence. Future clinical assessment should prioritize behavioral metrics, specifically poly-use intensity and product strength.

Strengths and limitations

This investigation aimed to represent the initial application of a person-centered LCA to ONP consumption within the Gulf Cooperation Council region. Such an approach offers a distinct methodological progression beyond conventional variable-centered prevalence estimations. By deriving user phenotypes grounded in functional etiology rather than relying solely upon rudimentary usage frequencies, our analytical framework successfully delineated a concealed high-risk demographic that standard aggregate statistics frequently obscure. Furthermore, isolating the adult poly-product user profile potentially challenges prevailing Western-centric narratives that primarily conceptualize these novel products as youth initiation gateways. This localized contextualization provides valuable ecological validity that could directly inform targeted public health strategies in Saudi Arabia.

These findings must nevertheless be interpreted within the context of several methodological constraints. The cross-sectional nature of the study design inherently precludes the establishment of causal inferences regarding the longitudinal stability of the identified phenotypes. It remains uncertain whether the affect-regulating dual-use pattern constitutes a permanent functional state or merely represents a transitional phase preceding further toxicological escalation. Additionally, utilizing a non-probability purposive sampling strategy supplemented by snowball recruitment within the Jazan Province may restrict the broader generalizability of our estimates across the national population. Snowball sampling, in particular, risks compounding selection bias by channeling recruitment through existing social networks of users, which could inadvertently result in the oversampling of high-intensity or socially connected consumers. Furthermore, the near-exclusive male composition of the sample (97.4%) represents a substantial limitation, as it precludes any meaningful inference regarding female ONP use patterns, motivations, or adverse effect profiles. Future investigations should employ probability-based sampling frameworks and actively oversample female users to ensure more representative and generalizable findings. We also acknowledge that relying upon self-reported metrics introduces the potential for recall bias, even though the documented adverse effects align closely with established biological plausibility. Finally, while implementing the modified FTND was necessary to provide comparative baselines, this instrument demonstrated notable psychometric inadequacies. Its apparent insensitivity to the complexities of poly-product dependence underscores a critical vulnerability in current clinical assessment methodologies.

Future prospective

Subsequent scientific inquiry would likely benefit from transitioning away from cross-sectional observations toward rigorous longitudinal surveillance architectures. Such tracking is essential to ascertain the temporal durability of the affect-regulating phenotype while accurately quantifying the conversion rates of rational switchers into either sustained abstinence or entrenched dual-use. Given the apparent psychometric shortcomings of standard dependence indices observed within this cohort, researchers should prioritize validating assessment instruments explicitly designed to capture poly-product consumption dynamics. Frameworks calibrated specifically for modern consumption habits, potentially including the About-Dependence scale, might offer superior construct validity in these increasingly complex scenarios. These refined psychometric evaluations should ideally be conducted in tandem with specialized pharmacokinetic investigations that seek to correlate self-reported simultaneous usage behaviors with objective biological markers of systemic nicotine exposure, such as plasma cotinine concentrations.

## Conclusions

This investigation suggests that ONP consumption in Saudi Arabia may be best understood through three divergent user phenotypes, implying that clinical risk may be shaped primarily by user behaviors and functional motivations rather than inherent product properties alone. Although a rational switcher profile supports targeted harm reduction for a specific subset, the concerning prevalence of escalating poly-product users necessitates recalibrating public health strategies. Regulatory frameworks might benefit from moving beyond generalized prevalence monitoring to systematically addressing the chemical and behavioral determinants, such as high-pH formulations and aggressive flavor profiles, that potentially catalyze simultaneous product stacking. Clinically, the apparent inadequacy of standard dependence metrics in this novel context highlights a critical need for advanced behavioral profiling. Therapeutic interventions will likely require significant customization, distinguishing between the mitigation of underlying emotion dysregulation for affect-regulating consumers and the acute disruption of high-intensity consumption cycles for escalating phenotypes.

## Appendices

Appendix A: oral nicotine pouch (ONP) user survey instrument

This appendix details the comprehensive electronic survey instrument utilized for data collection. The questionnaire was structured to sequentially capture data across six distinct domains: screening and eligibility (Section 1), sociodemographic characteristics and baseline nicotine exposure (Section 2), detailed consumption behaviors and stacking habits (Section 3), physical dependence parameters (Section 4), sustained functional motivations (Section 5), and the incidence of acute adverse effects (Section 6). The instrument was subjected to expert panel validation and pilot testing prior to deployment to ensure construct validity and target population comprehension.

**Table 7:** Study Instrument ONPs, Oral Nicotine Pouches

Item No.	Question / Statement	Response Type
Section 1: Screening & Consent		
1	Have you used nicotine pouches (ONPs) in the past 30 days?	□ Yes / □ No
2	I voluntarily agree to participate in this study, understand that my responses are confidential, and I can withdraw at any time.	□ Yes / □ No
3	Are you 18 years or older?	□ Yes / □ No
Section 2: Sociodemographics & Nicotine Exposure		
4	What is your age?	Numerical Entry (e.g., 18–99)
5	What is your gender?	□ Male □ Female
6	What is the highest level of education you have completed?	Single-choice: ○ No formal education ○ Primary school ○ Secondary school ○ High school ○ Bachelor & Above
7	What is your current employment status?	Single-choice: ○ Student ○ Employed (full-time) ○ Employed (part-time) ○ Unemployed ○ Freelancer ○ Retired
8	What is your approximate monthly income (in SAR)?	Numerical Entry
9	Where do you currently reside?	Single-choice: ○ Urban area ○ Rural area
10	When did you first start using nicotine pouches?	Single-choice: ○ Within the past month ○ 1–6 months ago ○ 6–12 months ago ○ More than a year ago
11	Have you ever used any other nicotine-related product (besides ONPs)? (This question has conditional logic depending on its answer the following 4 questions are either answered or skipped)	□ Yes / □ No
12	At what age did you first use any nicotine-related product? (If “Yes” to Q11)	Numerical Entry (years)
13	If you currently use other nicotine products, what are they? [Multiple answers; comma-separated] (If “Yes” to Q11)	Checkboxes: □ Cigarettes □ E-cigarettes/Vapes □ Shisha (Waterpipe) □ Heated Tobacco (IQOS) □ Smokeless Tobacco (Snus, Chewing tobacco)
14	How often do you use the nicotine product(s) selected in Q13? (If “Yes” to Q11)	Single-choice: ○ Every day ○ A few times a week ○ A few times a month ○ Occasionally (less than once a month)
15	Which nicotine product do you consider your primary nicotine source? (If “Yes” to Q11)	Single-choice: ○ Nicotine pouches ○ Cigarettes ○ E-cigarettes/Vapes ○ Waterpipe/Shisha ○ Heated Tobacco (IQOS) ○ Smokeless Tobacco (Snus, Chewing tobacco)
Section 3: Consumption Behaviors		
16	How many days per week do you consume ONPs?	Categories: 1-7
17	On a typical day, how many nicotine pouches do you consume?	Numerical Entry (e.g., 5 pouches/day)
18	What nicotine strength do you primarily use?	□ 3 mg □ 6 mg □ 10 mg □ More than 10 mg
19	On average, how many minutes do you keep a nicotine pouch in your mouth?	Categories: 5-10 minutes 10-15 minutes 15-20 minutes 20-25 minutes + 30 minutes
20	What is the most common reason you remove a nicotine pouch?	□ Nicotine effect decreases □ Discomfort (burning, irritation) □ To eat/drink □ Flavor is gone □ Suggested usage time is reached □ Out of habit □ Other: __________
21	What is the highest number of nicotine pouches you have used at the same time?	Categories: 1-10
22	How often do you use multiple nicotine pouches at the same time?	□ Never □ Occasionally (once or twice a month) □ Frequently (weekly) □ Regularly (almost daily)
23	In which situations do you most frequently feel the urge to use a nicotine pouch? (Select all that apply.)	□ Stress/anxiety □ Boredom/need oral fixation □ Habitual routine (post-meal, commute, etc.) □ Studying/working/focus □ Social settings □ With caffeine □ Craving nicotine (prefer ONPs) □ Hunger/appetite suppression □ Other: __________
24	Over the past six months, how has your nicotine pouch use changed?	□ Increased (more frequent) □ Decreased (less frequent) □ Stayed the same
25	What factors influenced this change in your ONP use? (Select all that apply.)	□ Stronger preference over other products □ Needed stronger/more frequent doses □ Health concerns □ Cost □ Switched to other products □ Social environment changes □ Other: __________
Section 4: FTND-ST Adapted to ONP		
Section 5: Motivations for Nicotine Pouch Use Scale		
32	ONPs give me a stronger nicotine effect than other products I have tried.	Positive
33	ONPs help me manage stress, relax, or improve my mood.	Positive
34 (Reverse)	Over time, I have started to feel that ONPs are unnecessary for me.	Negative
35	ONPs are my preferred nicotine product because they are discreet and easy to use anywhere.	Positive
36	I continue using ONPs because I see many people around me using them.	Positive
37 (Reverse)	I could stop using ONPs anytime I wanted to, without any difficulty.	Negative
38	I believe ONPs are safer than other nicotine products, so I feel no reason to stop.	Positive
39 (Reverse)	If ONPs were no longer available, I would not actively look for another nicotine product.	Negative
40	I have switched to ONPs because they helped me stop using other tobacco products.	Positive
Section 6: Adverse Effects		
41a	Dizziness or lightheadedness	□ 1 □ 2 □ 3 □ 4 □ 5
41b	Nausea or stomach discomfort	□ 1 □ 2 □ 3 □ 4 □ 5
41c	Heart palpitations or rapid heartbeat	□ 1 □ 2 □ 3 □ 4 □ 5
41d	Headache	□ 1 □ 2 □ 3 □ 4 □ 5
41e	Sweating or feeling clammy	□ 1 □ 2 □ 3 □ 4 □ 5
41f	Insomnia or difficulty sleeping	□ 1 □ 2 □ 3 □ 4 □ 5
41g	Anxiety or restlessness	□ 1 □ 2 □ 3 □ 4 □ 5
41h	Burning sensation or gum irritation	□ 1 □ 2 □ 3 □ 4 □ 5
41i	Loss of taste or changes in taste perception	□ 1 □ 2 □ 3 □ 4 □ 5
42	Have you noticed that these symptoms appear specifically after using nicotine pouches?	□ No direct link □ Yes, within minutes □ Yes, gradually after multiple pouches □ Yes, only with high-strength ONPs □ Yes, only with multiple pouches □ Other: _________
43	Have you modified your ONP consumption due to experiencing these symptoms?	□ No, I continue as usual □ Yes, reduced frequency/dosage □ Yes, switched to lower nicotine strength □ Yes, stopped using ONPs □ Other: _________

Item response structures across the instrument were optimized to capture varying degrees of behavioral and physiological phenomena. Responses within the sustained functional motivations domain (Section 5) are captured using declarative items designed to evaluate both positive and negative psychological reinforcement mechanisms. Furthermore, the incidence and severity of acute physiological adverse effects (Section 6), such as localized mucosal irritation and autonomic symptoms, are quantified utilizing a standardized 5-point frequency scale, enabling a characterization of subjective tolerability profiles among users.

To measure physiological dependence (Section 4), the instrument utilizes the Fagerström Test for Nicotine Dependence-Smokeless Tobacco (FTND-ST; Ebbert et al., 2006 [24]). For the purposes of this study, the tool was contextually adapted to the target population by substituting the term "smokeless tobacco" with "oral nicotine pouches" across all relevant prompts. This modification does not fundamentally alter the underlying psychological construct. To ensure strict adherence to publisher copyright and licensing terms, the specific text of the adapted FTND-ST items is not reproduced within this document. However, the operational scoring mechanics remain strictly identical to standard diagnostic protocols, utilizing a combination of binary (0-1) and four-point (0-3) weighted scales to generate a validated, composite dependence severity index.
